# miR-136-5p Preferentially Suppresses Cancer Stem-like Cells in Pancreatic Cancer

**DOI:** 10.3390/ijms27083686

**Published:** 2026-04-21

**Authors:** Hiroyuki Yamamoto, Yuhki Yokoyama, Shihori Kouda, Ruijia Yang, Yingjue Zhang, Jiaqi Wang, Yoshihiro Morimoto, Tsuyoshi Hata, Akira Inoue, Daisuke Okuzaki, Naotsugu Haraguchi, Hidekazu Takahashi, Satoshi Shibata, Hirofumi Yamamoto, Masaki Mori

**Affiliations:** 1 Department of Molecular Pathology, Division of Health Sciences, Graduate School of Medicine, The University of Osaka, Suita 565-0871, Japan; yamamoto.hiroyuki.eah@osaka-u.ac.jp (H.Y.); hyamamoto@sahs.med.osaka-u.ac.jp (H.Y.); 2 Department of Surgery, Gastroenterological Surgery, Graduate School of Medicine, The University of Osaka, Suita 565-0871, Japan; 3 Department of Gastroenterological Surgery, Osaka General Medical Center, Osaka 558-8558, Japan; 4 Department of Gastroenterological Surgery, Ikeda City Hospital, Ikeda 563-8510, Japan; 5 Laboratory for Human Immunology (Single Cell Genomics), WPI Immunology Frontier Research Center, The University of Osaka, Suita 565-0871, Japan; 6 Department of Gastroenterological Surgery, Kindai University Nara Hospital, Ikoma 630-0293, Japan; 7 Department of Gastroenterological Surgery, Osaka International Medical and Science Center, Osaka Keisatsu Hospital, Osaka 543-0042, Japan; 8 Graduate School of Medicine, Tokai University, Isehara 259-1193, Japan

**Keywords:** pancreatic cancer, cancer stem-like cells, miR-136-5p, DCLK1, miRNA-based therapy, drug sensitivity

## Abstract

In pancreatic cancer, cancer stem-like cells (CSCs) contribute to tumor initiation, reduced drug sensitivity, and recurrence. Limited strategies are currently available to target this cell population. Here we used a proteasome-low CSC enrichment system to identify microRNAs that negatively regulate CSC-like properties. From PANC-1 cells expressing a ZsGreen–ODC degron reporter, a proteasome-low population was isolated through sequential fluorescence-activated cell sorting of ZsGreen-positive cells. Molecular and functional analyses confirmed the CSC-like phenotype of this cell population. Integrated in silico analysis was used to select 31 microRNAs predicted to target CSC-related molecules, which were then evaluated by in vitro viability-based screening to identify candidates that selectively suppressed the viability of CSC-like cells, relative to non-CSCs. Moreover, comprehensive miRNA expression profiling revealed that miR-136-5p was downregulated in the CSC-like population and was therefore selected for further analysis. Mechanistically, miR-136-5p directly targets the 3′ untranslated region of DCLK1 and reduces its expression, with a greater reduction in the short isoform. Finally, in a CSC-derived xenograft mouse model, systemic delivery of miR-136-5p using super carbonate apatite nanoparticles significantly suppressed tumor growth. Taken together, these findings suggest that miR-136-5p restoration may provide a therapeutic approach for targeting CSC-driven tumor growth in pancreatic cancer.

## 1. Introduction

Pancreatic ductal adenocarcinoma (PDAC), the predominant histological type of pancreatic cancer, is a highly aggressive malignancy and was responsible for 467,005 cancer deaths worldwide in 2022 [1]. Despite advances in diagnostic imaging, surgical techniques, and systemic therapies, its prognosis remains extremely poor [2,3]. For resectable disease, the standard treatment consists of curative surgical resection followed by adjuvant therapy, with modified FOLFIRINOX providing the greatest survival benefit in fit patients [2,3]. For unresectable disease, standard systemic treatment includes gemcitabine plus nab-paclitaxel or FOLFIRINOX, and Poly(ADP-ribose) polymerase (PARP) inhibitors are used in selected patients [2,4]. However, the overall therapeutic benefit remains limited, and the 5-year survival rate is still approximately 12% [2,5]. These clinical features highlight the urgent need for novel therapeutic strategies for pancreatic cancer. Increasing evidence suggests that these clinical features cannot be fully explained by the behavior of the bulk tumor alone, but rather reflect functional heterogeneity driven by distinct cellular states within pancreatic cancer [4,6,7].

Cancer stem-like cells (CSCs) have been proposed to represent a cellular state that sustains high tumorigenicity, drives therapeutic resistance, and contributes to disease relapse in multiple cancer types, including pancreatic cancer [8,9,10,11]. CSC-like cells in pancreatic cancer have been identified using cell surface markers, such as CD44, CD133, and CD44v9 [12,13,14], as well as functional assays, including sphere formation and drug resistance [7,9,14,15]. However, CSC-associated marker expression is highly plastic, and strongly influenced by cellular context and microenvironmental cues, such that marker-based CSC identification is inherently controversial [10]. These limitations highlight the need for functional approaches based on intrinsic cellular properties [6].

Low proteasome activity has emerged as a functional hallmark of CSC-like cell states, which has been associated with enhanced stress tolerance and increased tumor-initiating capability [16,17,18]. The ZsGreen–ornithine decarboxylase (ODC) degron reporter system enables the visualization and isolation of cells with low proteasome activity, providing a marker-independent approach to enriching CSC-like cells [19,20]. This system has been applied in several different malignancies, including pancreatic cancer [9,20].

MicroRNAs (miRNAs) are key post-transcriptional regulators of gene expression, which play crucial roles in cancer progression. Several miRNAs reportedly regulate CSC-associated signaling pathways (including Notch and Wnt/β-catenin signaling), and influence stem-like phenotypes [21,22,23,24,25,26]. Because miRNAs can simultaneously modulate multiple target genes and pathways, they are attractive therapeutic candidates for heterogeneous cancers such as pancreatic cancer. However, the clinical translation of miRNA therapy remains challenging, largely because of difficulties in achieving safe and efficient systemic delivery to tumor tissues [27,28]. DCLK1 is a regulator of cancer stemness and tumor progression. It has been implicated in CSC-related phenotypes in pancreatic cancer and has been identified as a target of miRNA-mediated regulation [29,30,31,32,33]. However, it remains unclear which miRNAs preferentially regulate functionally defined pancreatic CSC-like cell states, and how they contribute to the maintenance of CSC phenotypes.

Super carbonate apatite (sCA) is a non-viral, pH-sensitive inorganic nanoparticle system developed for systemic delivery of siRNA/miRNA. This platform facilitates endosomal release of incorporated nucleic acids and has shown efficient in vivo tumor delivery with relatively low accumulation in normal tissues in previous studies [34,35]. Because systemic delivery remains a major challenge in miRNA-based therapy, sCA was selected in the present study as the delivery platform for in vivo evaluation.

In the present study, we aimed to identify miRNAs that suppress pancreatic CSC-like cell states, defined by low proteasome activity. To this end, we integrated a ZsGreen–ODC degron-based functional CSC enrichment system with in silico prediction, functional screening, and expression profiling of candidate miRNAs. We further evaluated the biological and therapeutic relevance of the candidate miRNAs in both in vitro models and an in vivo xenograft model, in the latter setting using an sCA nanoparticle formulation for systemic delivery of miR-136-5p.

## 2. Results

### 2.1. Establishment of CSC-like Cell Population with Low Proteasome Activity Using the ZsGreen–ODC Degron System

**Scheme 1:**
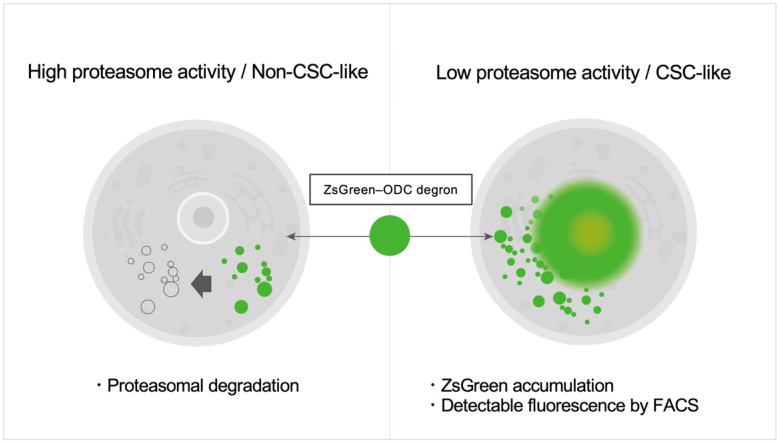
Schematic illustration of the ZsGreen–ODC degron reporter system. In cells with high proteasome activity, the reporter is degraded and fluorescence is lost, whereas in cells with low proteasome activity, the reporter accumulates and is detectable by FACS. Green circles indicate retained signal, dotted circles indicate signal loss due to proteasomal degradation, and arrows indicate the process shown.

To establish a pancreatic cancer stem-like cell (CSC-like) model, we introduced a ZsGreen–ODC degron reporter system into PANC-1 cells. In this model, the ODC degron sequence directs ZsGreen for proteasomal degradation under high proteasome activity, while cells with reduced proteasome activity retain and accumulate ZsGreen fluorescence (Scheme 1).

#### 2.1.1. Establishment of ZsGreen^−^ Cells and Enriched ZsGreen^+^ Cells from a Pancreatic Cancer Cell Line

**Figure 1:**
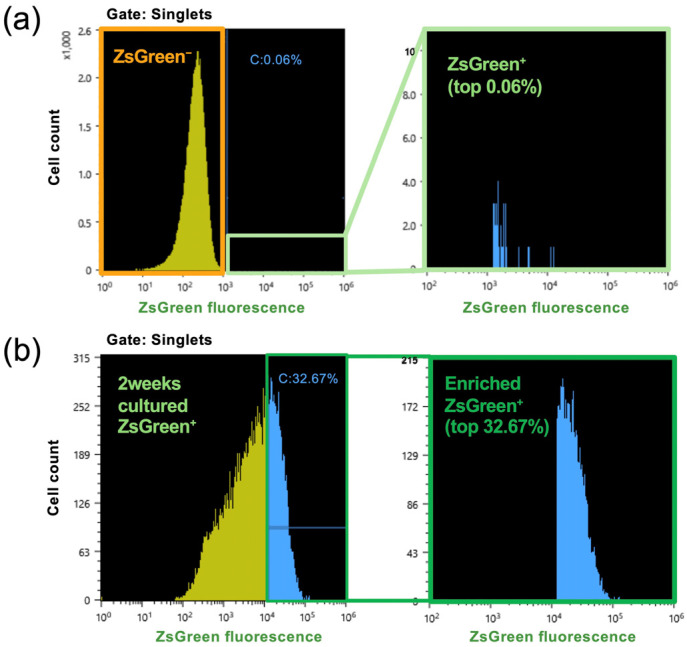
Establishment of a low-proteasome activity cell population using the ZsGreen–ODC degron system. (**a**) Initial sorting of ZsGreen^−^ and ZsGreen^+^ cells. (**b**) Secondary sorting to obtain Enriched ZsGreen^+^ cells.

PANC-1 cells expressing the ZsGreen–ODC degron reporter were analyzed by flow cytometry. Substantial ZsGreen fluorescence was detected in only a small subset of cells (0.06%), while the majority of cells exhibited little or no fluorescence, consistent with high proteasome activity in most cells (Figure 1a). The ZsGreen-negative population was collected and defined as ZsGreen^−^ cells. Cells exhibiting the top 0.06% of ZsGreen fluorescence intensity were isolated by fluorescence-activated cell sorting (FACS), and cultured. After ZsGreen^+^ cells were expanded for 2 weeks, highly fluorescent cells (top 32.67%) were re-sorted and defined as Enriched ZsGreen^+^ cells (Figure 1b). ZsGreen^−^ and Enriched ZsGreen^+^ cells were used for all subsequent analyses. Supplementary Figures S1 and S2 show representative images and the detailed gating strategy.

#### 2.1.2. Functional Evaluation of CSC-like Phenotypes Among ZsGreen^−^ and Enriched ZsGreen^+^ Cells

**Figure 2:**
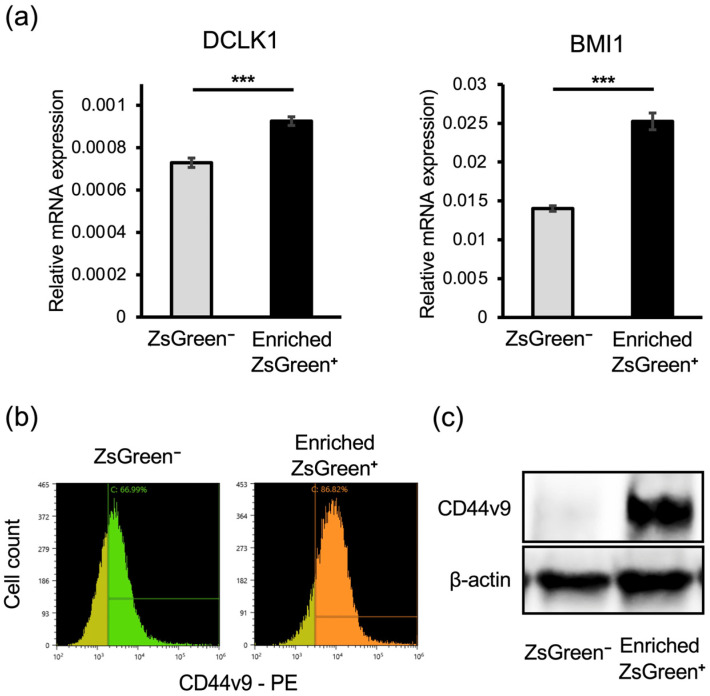
CSC-associated molecular features of Enriched ZsGreen^+^ cells. (**a**) Relative mRNA expressions of DCLK1 and BMI1. Data are presented as mean ± SD (*n* = 4). *** *p* < 0.001. (**b**) Representative flow cytometric histograms of CD44v9 expression in ZsGreen^−^ and Enriched ZsGreen^+^ cells. The MFI values were 8445 for ZsGreen^–^ cells and 18,891 for Enriched ZsGreen^+^ cells. (**c**) Western blot analysis of CD44v9 protein expression.

Quantitative RT–PCR analysis revealed that the expression levels of CSC-associated genes, including DCLK1 and BMI1 [30,32,36], were significantly higher in Enriched ZsGreen^+^ cells, compared with ZsGreen^−^ cells (Figure 2a). Representative flow cytometric analysis showed higher CD44v9 expression in Enriched ZsGreen^+^ cells than in ZsGreen^−^ cells, with mean fluorescence intensity (MFI) values of 18,891 and 8445, respectively (Figure 2b). Consistently, Western blot analysis confirmed that CD44v9 protein expression was elevated in Enriched ZsGreen^+^ cells, and was barely detected in ZsGreen^−^ cells (Figure 2c).

**Figure 3:**
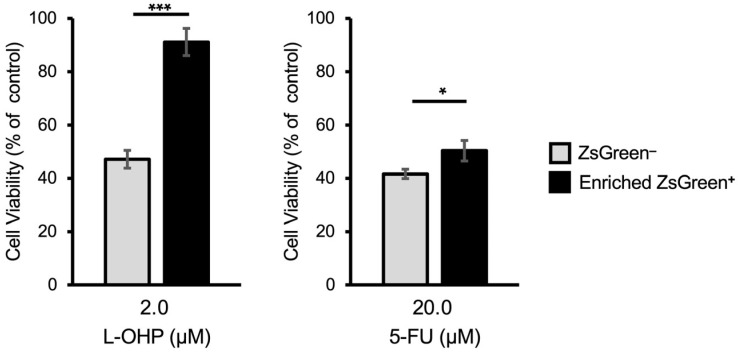
Reduced sensitivity of Enriched ZsGreen^+^ cells to L-OHP and 5-FU. Cell viability of Enriched ZsGreen^+^ and ZsGreen^−^ cells after 72 h treatment with L-OHP (2.0 µM) or 5-FU (20.0 µM). The drug concentrations of both L-OHP and 5-FU were selected based on the IC_50_ values determined in ZsGreen^−^ cells. Viability was normalized to the corresponding vehicle-treated control within each cell fraction. Data are presented as mean ± SD (*n* = 4). *** *p* < 0.001; * *p* < 0.05.

Since CSC-like cells have often been associated with reduced drug sensitivity [7,37], we also evaluated the responses of ZsGreen^−^ and Enriched ZsGreen^+^ cells to oxaliplatin (L-OHP) and 5-fluorouracil (5-FU). After 72 h of treatment with 2 µM L-OHP, Enriched ZsGreen^+^ cells maintained significantly higher viability than ZsGreen^−^ cells (Figure 3, *** *p* < 0.001). Similarly, after 72 h of treatment with 20 µM 5-FU, Enriched ZsGreen^+^ cells showed significantly higher viability compared with ZsGreen^−^ cells (Figure 3, * *p* < 0.05).

**Figure 4:**
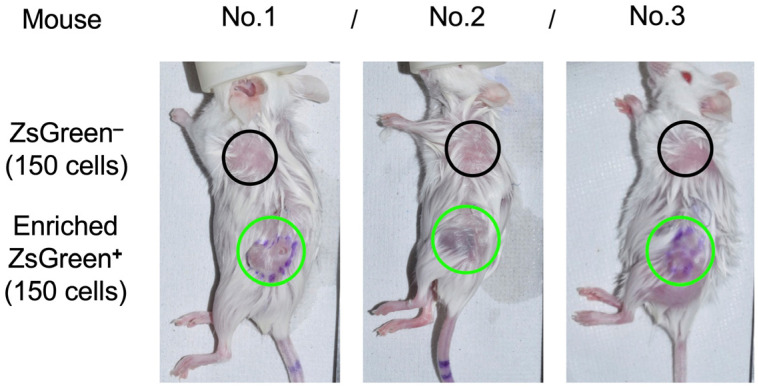
Tumor-forming capacity of CSC-enriched cells in vivo. Tumor formation after subcutaneous transplantation of 150 ZsGreen^−^ or Enriched ZsGreen^+^ cells into SCID beige mice (*n* = 3 per group). Black circles indicate the sites transplanted with ZsGreen^−^ cells, and green circles indicate the sites transplanted with Enriched ZsGreen^+^ cells.

We next assessed tumor-forming capacity under limiting cell conditions. A total of 150 cells were subcutaneously injected into SCID beige mice. After 42 days, tumors had developed in all mice transplanted with Enriched ZsGreen^+^ cells (3/3), whereas no tumors were observed in mice that received ZsGreen^−^ cells (0/3). In the Enriched ZsGreen^+^ group, the tumors progressively enlarged and exceeded 10 mm in diameter during the observation period (Figure 4). These results are consistent with a functionally defined CSC-like phenotype.

### 2.2. Identification of miRNAs That Selectively Suppress the CSC-like Cell Population

#### 2.2.1. In Silico Selection of Candidate miRNAs

**Scheme 2:**
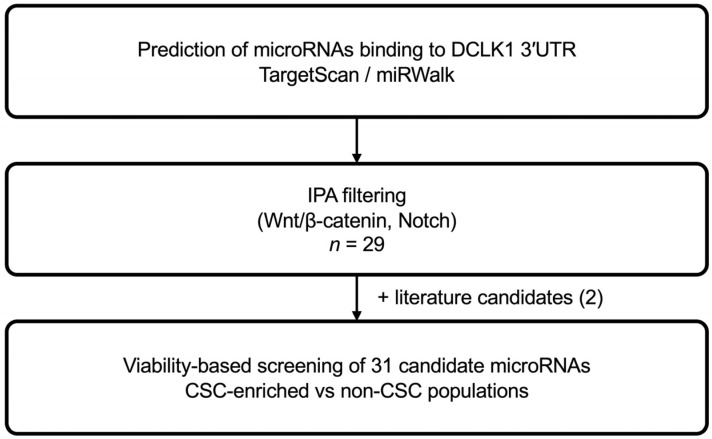
Process for identifying candidate miRNAs for functional screening. MicroRNAs predicted to target the DCLK1 3′ UTR were identified using TargetScan and miRWalk. Further prioritization based on reported involvement in the stemness-related Notch and Wnt/β-catenin signaling pathways using Ingenuity Pathway Analysis (IPA) yielded 29 candidates, and two additional microRNAs supported by literature evidence were included, resulting in 31 candidate miRNAs.

To identify miRNAs involved in CSC-like properties, we first performed in silico prediction (Scheme 2). We used TargetScan Human 7.0 and miRWalk 3.0 to identify miRNAs predicted to target the 3′ untranslated region (3′ UTR) of the putative CSC-associated molecule DCLK1 [30,32]. Details of the selected candidate miRNAs and the basis for their selection are provided in Supplementary Table S1. Next, these candidates were further prioritized based on their reported involvement in the stemness-related Notch and Wnt/β-catenin signaling pathways [25,26]. Together with two additional candidates selected from the literature, this process yielded a focused set of 31 miRNAs for functional screening.

#### 2.2.2. Viability-Based Functional Screening

**Figure 5:**
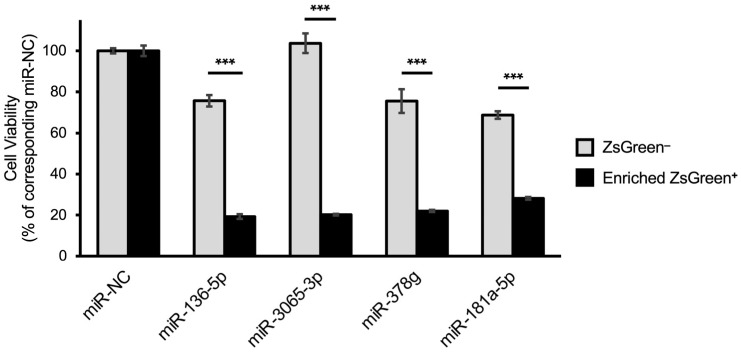
Validation of the preferential suppression of Enriched ZsGreen^+^ cells by candidate miRNAs. At 48 h after transfection with the indicated miRNA mimics (30 nM), the cell viability of ZsGreen^−^ and Enriched ZsGreen^+^ cells was assessed and expressed as % of the corresponding miR-NC within each cell fraction. miR-NC was set to 100% separately for ZsGreen^−^ and Enriched ZsGreen^+^ cells. Data are presented as mean ± SD (*n* = 4). *** *p* < 0.001.

To perform viability-based screening of the 31 prioritized candidates, miRNA mimics (30 nM) were transfected into ZsGreen^−^ and Enriched ZsGreen^+^ cells. At 48 h after transfection, all miRNAs reduced the viability of ZsGreen^−^ cells to varying extents (34.5–74.1% of the medium-only control), and 15 miRNAs showed greater suppression in Enriched ZsGreen^+^ cells, compared with ZsGreen^−^ cells (Supplementary Table S2, Rank 1–15). Notably, four miRNAs—miR-136-5p, miR-3065-3p, miR-378g, and miR-181a-5p—exhibited the greatest preferential suppression in Enriched ZsGreen^+^ cells. These miRNAs reduced viability to <10% of the medium-only control, and yielded selectivity ratios (cell viability of Enriched ZsGreen^+^/ZsGreen^−^) ranging from 0.12 to 0.26 (Supplementary Table S2). Thus, these four candidates were selected for validation. Under identical conditions, all four of these miRNAs significantly suppressed viability in Enriched ZsGreen^+^ cells (*p* < 0.01 for miR-378g; *p* < 0.001 for the others) (Figure 5). To further illustrate the prediction basis for these candidate miRNAs, detailed TargetScan prediction information is provided in Supplementary Figure S3.

#### 2.2.3. Comprehensive miRNA Expression Analysis of ZsGreen^−^ and Enriched ZsGreen^+^ Cells by nCounter

**Figure 6:**
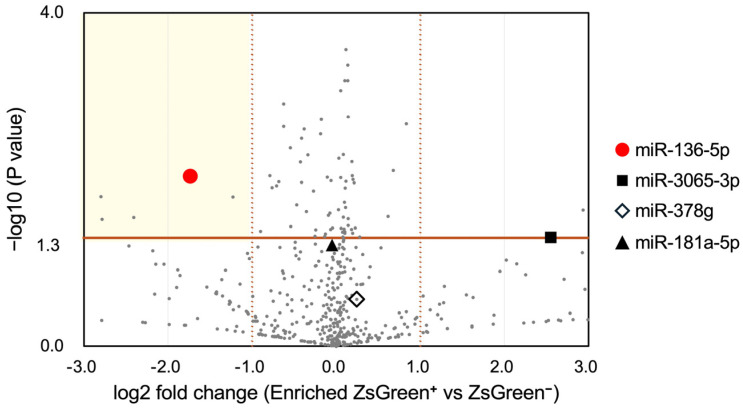
Volcano plot showing differential miRNA expression between Enriched ZsGreen^+^ and ZsGreen^–^ cells. Among the four candidate miRNAs, miR-136-5p exhibited the largest decrease in Enriched ZsGreen^+^ cells. Thresholds were set at |log2 fold change| ≥ 1 and −log10(*p*) ≥ 1.3.

To assess miRNA expression in ZsGreen^−^ and Enriched ZsGreen^+^ cells, we performed comprehensive miRNA profiling using the NanoString nCounter system. Among the four candidate miRNAs, miR-136-5p was markedly downregulated in Enriched ZsGreen^+^ cells compared with ZsGreen^−^ cells, with a log2 fold change of −1.734 and a *p* value of 0.009 (Figure 6). The global miRNA expression profile is shown in Supplementary Figure S4. Based on its preferential suppression in functional screening and its reduced expression in the CSC-enriched fraction, miR-136-5p was selected for subsequent mechanistic analyses.

### 2.3. miR-136-5p Targets DCLK1 and Suppresses DCLK1 Protein Expression

**Figure 7:**
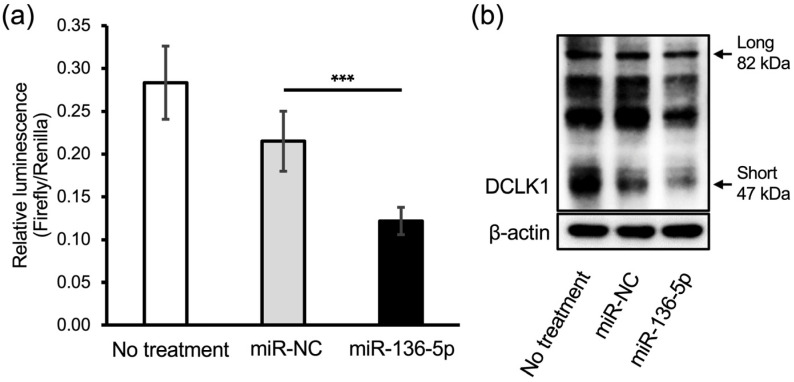
miR-136-5p suppresses DCLK1 and reduces the short isoform of DCLK1. (**a**) A dual-luciferase reporter assay was performed using HCT116 cells co-transfected with miR-136-5p or miR-NC (50 nM), and a pmirGLO reporter vector (25 ng) containing a fragment of the DCLK1 3′ UTR encompassing the predicted miR-136-5p binding site. Firefly luciferase activity was normalized to Renilla luciferase activity. No treatment indicates transfection of the pmirGLO reporter vector alone. Data are presented as mean ± SD (*n* = 6). *** *p* < 0.001. (**b**) Western blot analysis of the DCLK1 long (82 kDa) and short (47 kDa) isoforms at 48 h after transfection with miR-136-5p or miR-NC (30 nM). ACTB served as a loading control.

To elucidate the mechanism underlying the suppression of CSC-enriched cell viability by miR-136-5p, we used a dual-luciferase reporter assay to determine whether DCLK1 is a direct target of miR-136-5p. Transfection with a miR-136-5p mimic significantly reduced the luciferase activity of a DCLK1 3′ UTR reporter construct, compared with transfection with miR-NC (*p* < 0.001) (Figure 7a). Furthermore, Western blot analysis showed that transfection with the miR-136-5p mimic reduced DCLK1 protein expression, with a greater reduction in the short isoform (47 kDa) than in the long isoform (82 kDa) (Figure 7b).

### 2.4. Systemic Delivery of miR-136-5p Suppresses CSC-Derived Tumor Growth In Vivo

**Figure 8:**
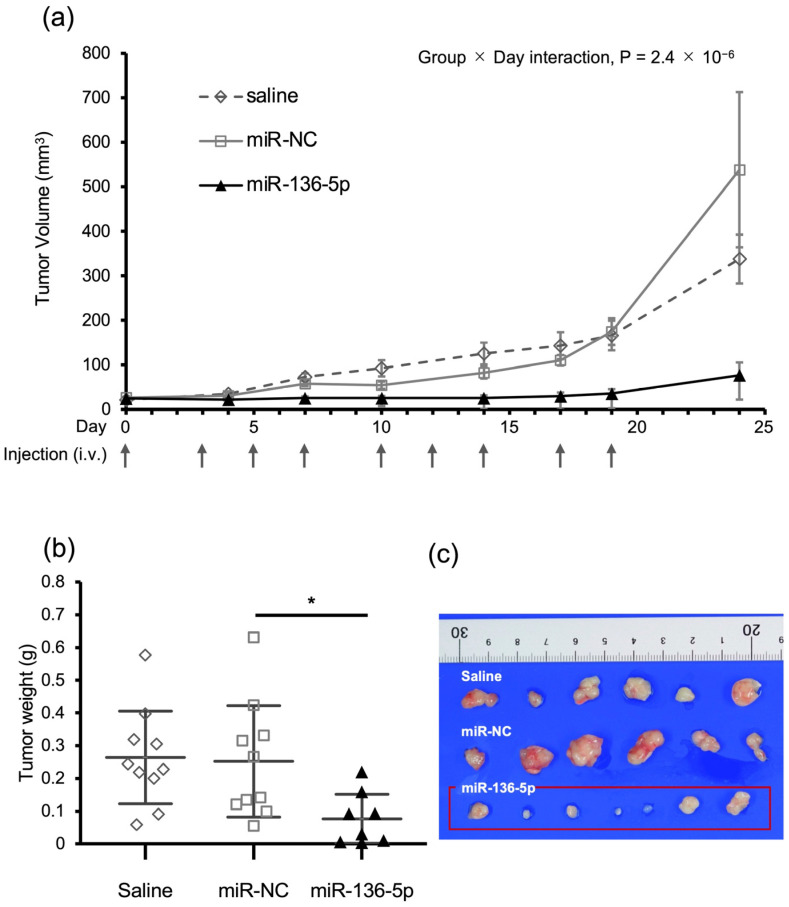
Systemically delivered miR-136-5p reduces CSC-derived tumor growth. (**a**) Tumor volume over time in Enriched ZsGreen^+^ PANC-1 xenografts treated with saline (*n* = 5), miR-NC (*n* = 5), or miR-136-5p (*n* = 4). miR-136-5p or miR-NC (40 µg per injection) was formulated with sCA, and intravenously administered on the days indicated by arrows. Data are shown as mean ± SD. Tumor growth was analyzed using a linear mixed-effects model, revealing a significant Group × Day interaction (*p* = 2.4 × 10^−6^). Post-hoc Tukey-adjusted comparisons of tumor growth slopes demonstrated significantly slower tumor growth in the miR-136-5p group, compared with the saline and miR-NC groups (both *p* < 0.0001), while no difference was observed between the saline and miR-NC groups (*p* = 0.915). (**b**) Tumor weights on day 24. Each symbol represents an individual tumor. Bars indicate mean ± SD. * *p* < 0.05 versus miR-NC (one-way ANOVA with Dunnett’s test). (**c**) Representative images of excised tumors on day 24.

To evaluate the therapeutic relevance of miR-136-5p, we examined the effect of systemic administration of miR-136-5p formulated with sCA (a non-viral miRNA delivery system [34,35]) in a xenograft model established from CSC-enriched ZsGreen^+^ PANC-1 cells (experimental design shown in Supplementary Figure S5). In this therapeutic model, tumor growth was significantly suppressed in the miR-136-5p–treated group compared with the saline and miR-NC control groups (Figure 8a). The tumor growth trajectories differed among these groups (*p* = 2.4 × 10^−6^, Group × Day interaction, linear mixed-effects model), with slower growth in the miR-136-5p group, compared with both control groups (*p* < 0.0001, post-hoc Tukey-adjusted comparisons). At the study end-point (day 24), tumor weight was significantly lower in the miR-136-5p group compared with the miR-NC group (*p* < 0.05, one-way ANOVA followed by Dunnett’s test) (Figure 8b). No significant difference was observed between the saline and miR-NC groups. Figure 8c shows representative images of excised tumors. Body weight remained stable across groups throughout the study (Supplementary Figure S6).

## 3. Discussion

In this study, we identified miR-136-5p as a microRNA that preferentially suppresses pancreatic cancer stem-like cells. This was achieved by integrating a proteasome activity-based functional CSC enrichment system with in silico prioritization, viability-based screening, and miRNA expression profiling. The ZsGreen–ODC degron system was used to enrich a ZsGreen^+^ fraction characterized by low proteasome activity. This population exhibited CSC-associated phenotypes—including increased expression of CSC markers, such as DCLK1 and BMI1; resistance to chemotherapy; and enhanced tumorigenicity. Restoration of miR-136-5p selectively reduced the viability of these CSC-enriched cells in vitro, and significantly suppressed tumor growth in vivo in a therapeutic experiment using a CSC-derived PANC-1 xenograft model.

A key feature of this study is the application of the ZsGreen–ODC degron reporter system, which enabled visualization and isolation of cells with low proteasome activity. Previous studies have demonstrated that cancer cells with low proteasome activity across multiple human malignancies—including colorectal, lung, pancreatic, and liver cancers—exhibit stem cell-like properties, such as elevated stem cell marker expression, increased resistance to chemotherapy and radiation, and enhanced tumorigenicity [9,16,17,18,38,39]. ZsGreen-labeled proteasome-low cells are typically rare, comprising < 1.0% of the total cell population in most cell types. Therefore, we previously established an enrichment strategy that combines cell sorting with subsequent culture expansion, and we demonstrated its clinical relevance [19]. In the present study, this approach enabled the establishment of Enriched ZsGreen^+^ cells, and facilitated the elucidation of mechanisms contributing to the pancreatic CSC-like state.

We used this platform to identify miR-136-5p. In silico analysis of miRNAs predicted to target DCLK1, and involved in CSC-related networks associated with either Notch or Wnt signaling, was integrated with comprehensive miRNA expression profiling using the nCounter system. We found that miR-136-5p was downregulated in Enriched ZsGreen^+^ cells. Moreover, miR-136-5p restoration resulted in a more pronounced suppression of proliferation among CSC-like Enriched ZsGreen^+^ cells. Although growth inhibition was also observed in ZsGreen^−^ cells, the magnitude of suppression was significantly greater in the CSC-like population, suggesting that the CSC compartment may be particularly vulnerable to miR-136-5p. This preferential inhibitory effect may provide a therapeutic advantage, by selectively targeting CSCs. Consistently, we found that systemic administration of miR-136-5p in xenograft models established from Enriched ZsGreen^+^ cells significantly suppressed tumor growth in vivo. These findings contrast with our previous report, which showed that miR-491-3p suppressed the growth of both ZsGreen^−^ and Enriched ZsGreen^+^ cells in a colorectal cancer CSC model [19]. While miR-136-5p has been reported as a tumor-suppressive miRNA in several human cancers [40,41], its preferential suppressive role in pancreatic CSC-like cells has not previously been demonstrated.

DCLK1 has been widely implicated in cancer stemness and tumor progression across multiple malignancies [30,31,42]. In the present study, we demonstrated that miR-136-5p directly targeted the 3′ UTR of DCLK1 and reduced DCLK1 protein expression. Moreover, Western blot analysis revealed a more pronounced decrease in the short isoform. Accumulating evidence supports the biological and clinical relevance of isoform-specific expression of DCLK1 [43,44]. In colorectal cancer, compared with the long isoform, the short isoform has been more closely linked to tumor progression, invasive potential, and poor patient outcome [44,45,46]. Because the short isoform lacks the N-terminal doublecortin domains, it may differ from the long isoform in subcellular behavior and biological function [43,46]. In this context, the greater reduction in the short isoform observed in the present study may be biologically relevant. Although the isoform-specific functions and regulatory mechanisms of DCLK1 in pancreatic cancer remain incompletely understood, our findings raise the possibility that miR-136-5p–mediated suppression of the short isoform may contribute to attenuation of CSC-like properties in pancreatic cancer cells.

Despite growing interest in miRNA-based therapeutics, clinical translation remains challenging due to the limited availability of safe and efficient systemic delivery systems [27]. While non-viral nanoparticles are generally considered safer than viral vectors, early clinical trials of the liposomal miR-34a mimic MRX34 revealed immune-related adverse events, leading to early termination of the study [28]. These experiences highlight the need for delivery platforms that enable systemic administration with reduced toxicity. Super carbonate apatite (sCA) is a pH-sensitive non-viral carrier capable of delivering nucleic acids, including miRNAs and siRNAs, which is designed to facilitate systemic administration with minimal immune activation [34,35]. In the present study, miR-136-5p formulated with sCA was systemically administered in vivo, and significantly suppressed the growth of tumors derived from Enriched ZsGreen^+^ cells, without observable adverse effects. Together with previous reports demonstrating the safety and efficiency of sCA-based formulations [47,48], our findings support the feasibility of combining CSC-directed miRNA therapy with non-viral delivery strategies in pancreatic cancer.

Several limitations of this study should be acknowledged. First, the present study was conducted using a single pancreatic cancer cell line (PANC-1). Although the ZsGreen–ODC degron system has been validated across multiple malignancies, including pancreatic cancer [9,20], and PANC-1 represents a clinically relevant KRAS-mutant PDAC model, it will be important to perform validation in additional PDAC models with distinct genetic backgrounds, to determine the generalizability of miR-136-5p–mediated CSC suppression. Second, although Enriched ZsGreen^+^ cells showed reduced sensitivity to L-OHP and 5-FU in the present assay, this single-time-point viability assay does not by itself distinguish slow-cycling-associated tolerance from other mechanisms contributing to reduced drug sensitivity. In addition, because the miRNA screening was performed under a single condition (30 nM, 48 h), it remains possible that different candidate miRNAs might have been identified under other experimental conditions, such as different concentrations or time points. Third, although the biological significance of the short isoform of DCLK1 has been reported in colorectal cancer, its precise role in pancreatic cancer remains unclear. Further functional studies are required to clarify the isoform-specific roles of DCLK1 in CSC maintenance in pancreatic cancer. Fourth, in vivo efficacy was evaluated using a subcutaneous xenograft model, which does not fully recapitulate the pancreatic tumor microenvironment. There remains a need for evaluation in an orthotopic transplantation model, to assess therapeutic efficacy in a more physiologically relevant context. Fifth, although the luciferase reporter assay supported targeting of the DCLK1 3′ UTR by miR-136-5p, confirmation using a mutant 3′ UTR reporter construct was not performed in the present study.

In summary, here we identified miR-136-5p as a microRNA that preferentially suppresses pancreatic cancer stem-like cells enriched by a proteasome activity-based functional model. Reduced expression of miR-136-5p may contribute to the maintenance of DCLK1-associated CSC features. Restoration of miR-136-5p, combined with a non-viral systemic delivery strategy, may provide a therapeutic approach for targeting CSC-driven tumor growth in pancreatic cancer.

## 4. Materials and Methods

### 4.1. Cell Lines and Cell Culture

Experiments were performed using the human pancreatic cancer cell line PANC-1, and the human colorectal cancer cell line HCT116. PANC-1 cells were used for the establishment and analysis of pancreatic cancer stem-like cells. HCT116 cells were used for luciferase reporter assays, due to their high transfection efficiency during co-transfection with plasmid DNA and miRNA [19]. These cell lines were obtained from the American Type Culture Collection (ATCC; Manassas, VA, USA), and were authenticated by ATCC documentation, morphological inspection, and mycoplasma testing. Both cell lines were cultured at 37 °C in a humidified atmosphere containing 5% CO_2_, in Dulbecco’s Modified Eagle’s Medium (DMEM; Nacalai Tesque, Kyoto, Japan) supplemented with 10% fetal bovine serum (Thermo Fisher Scientific, Waltham, MA, USA), 100 U/mL penicillin (Sigma-Aldrich, St. Louis, MO, USA; Merck KGaA, Darmstadt, Germany), and 100 µg/mL streptomycin (Nacalai Tesque).

### 4.2. ZsGreen–ODC Degron Reporter System and CSC Enrichment

Cells with low proteasome activity were identified using a ZsGreen–ornithine decarboxylase (ODC) degron reporter system, as previously described [17,19,20]. Briefly, the retroviral vector pQCXIN-ZsGreen-cODC was transfected into Platinum retroviral packaging cells (Cell Biolabs, San Diego, CA, USA), and then viral supernatants were used to infect PANC-1 cells. Stable reporter-expressing cells were established through selection with G418 (Geneticin; Sigma-Aldrich). Approximately 0.06% of cells were ZsGreen-positive (ZsGreen^+^), and the remaining cells were defined as ZsGreen^−^. ZsGreen^+^ cells were isolated by fluorescence-activated cell sorting (FACS) using a CellSorter SH800S (Sony Biotechnology Inc., San Jose, CA, USA). After sorting, ZsGreen^+^ cells were cultured and expanded. Two weeks later, cells exhibiting the highest ZsGreen fluorescence intensity (top 32.67%) were isolated and defined as Enriched ZsGreen^+^ cells. The ZsGreen^−^ and Enriched ZsGreen^+^ cells were used for subsequent experiments.

### 4.3. Flow Cytometry

Cells were harvested with 0.25% trypsin–EDTA solution (Nacalai Tesque), washed with phosphate-buffered saline (PBS; Nacalai Tesque), and then resuspended in staining buffer (PBS containing 2% fetal bovine serum). Flow cytometric analysis and cell sorting were performed using a CellSorter SH800S. Dead cells and debris were excluded based on forward and side scatter profiles, and singlet discrimination was performed by gating on FSC-H versus FSC-W. Fluorescence compensation was not necessary because only ZsGreen fluorescence was analyzed for sorting. Identical gating strategies were applied across experiments. For the analysis of cell surface markers, cells were incubated with the appropriate antibodies (listed in Supplementary Table S3) for 30 min at 4 °C. Isotype-matched antibodies were used as controls.

### 4.4. RNA Extraction and Quantitative RT-PCR

Total RNA was extracted using TRIzol Reagent (Thermo Fisher Scientific). From 1.0 µg total RNA, complementary DNA was synthesized using an oligo(dT) primer and a Reverse Transcription System (Promega, Madison, WI, USA). Quantitative RT-PCR was performed using LightCycler FastStart DNA Master SYBR Green I (Roche Diagnostics, Basel, Switzerland) or LightCycler TaqMan Master (Roche Diagnostics), on a LightCycler 2.0 system (Roche Diagnostics). Relative gene expression was calculated using the 2^−ΔΔCt^ method and normalized to GAPDH expression. Primer sequences are listed in Supplementary Table S4.

### 4.5. Western Blot Analysis

Cell lysates were prepared using RIPA buffer (FUJIFILM Wako Pure Chemical Corporation, Osaka, Japan) supplemented with protease inhibitors (Thermo Fisher Scientific). Proteins were subjected to 10% SDS-PAGE, and then transferred using the Trans-Blot Turbo system (Bio-Rad Laboratories, Hercules, CA, USA). Membranes were incubated with primary antibodies (listed in Supplementary Table S3), followed by horseradish peroxidase-conjugated secondary antibodies. Signals were detected using ECL substrate (Cytiva, Marlborough, MA, USA), and visualized with the ChemiDoc Touch Imaging System (Bio-Rad Laboratories).

### 4.6. Cell Proliferation Assay

Viable cells were quantified using the Cell Counting Kit-8 (Dojindo Molecular Technologies, Kumamoto, Japan), following the manufacturer’s instructions. The cells were incubated in the assay solution for 2 h, and then the number of viable cells in each well was determined by measuring the absorbance at 450 nm with a BIO-RAD Model 680 XR microplate reader (Bio-Rad Laboratories).

### 4.7. Drug Sensitivity Assay

ZsGreen^−^ and Enriched ZsGreen^+^ PANC-1 cells were seeded in 96-well plates, at 5000 cells per well, and then treated for 72 h with L-OHP (2.0 µM; Sigma-Aldrich) or 5-FU (20.0 µM; Sigma-Aldrich). Cell viability was assessed using Cell Counting Kit-8, and expressed relative to a vehicle-treated control. Drug concentrations were determined based on the IC_50_ values in ZsGreen^−^ cells, as obtained from preliminary dose–response experiments.

### 4.8. In Silico Identification of Candidate miRNAs

TargetScan Human 7.0 and miRWalk 3.0 were used to identify miRNAs predicted to bind the 3′ UTR of DCLK1. The predicted candidates were further filtered using Ingenuity Pathway Analysis (IPA; QIAGEN Digital Insights, Redwood City, CA, USA) with the default settings based on experimentally observed interactions, association with cancer, and involvement in stemness-related signaling pathways, including Notch and Wnt/β-catenin signaling. In addition, miRNAs supported by literature evidence were included, yielding a focused set of candidate miRNAs for functional screening. Details of the selected candidate miRNAs and the basis for their selection are provided in Supplementary Table S1.

### 4.9. miRNA Transfection

Synthetic miRNA mimics were purchased from Ajinomoto Bio-Pharma (Tokyo, Japan). Transfection was performed using Lipofectamine RNAiMAX (Thermo Fisher Scientific), following the manufacturer’s instructions. Cells were transfected with each miRNA mimic, or with negative control miRNA (miR-NC), at a final concentration of 30 or 50 nM. Supplementary Table S5 presents the miRNA sequences.

### 4.10. NanoString nCounter miRNA Expression Analysis

Total RNA (including small RNAs) was extracted from independently passaged ZsGreen^−^ and Enriched ZsGreen^+^ cells (*n* = 2 independent biological replicates per group). Global miRNA expression profiling was performed using the NanoString nCounter Human miRNA Expression Assay (NanoString Technologies, Seattle, WA, USA). Data processing and normalization were performed by the RIMD NGS Core Facility (Research Institute for Microbial Diseases/Immunology Frontier Research Center, The University of Osaka). Normalized count data—including fold change (FC) and log_2_ fold change (log2FC) values—were used for downstream analyses. The relative expression between groups was assessed using an unpaired two-tailed Welch’s *t*-test. Due to the limited number of biological replicates (*n* = 2 per group), these statistical analyses were considered exploratory. Volcano plots were generated using log2FC and −log_10_(*p* value), with exploratory thresholds set at |log2FC| ≥ 1 and −log_10_(*p*) ≥ 1.3 (corresponding to *p* ≤ 0.05).

### 4.11. pmirGLO Plasmid Vector Construction

A fragment of the DCLK1 3′ UTR (amplicon size, 414 bp) was amplified by PCR using the following primers: forward, 5′-TAGCCTCGAGTCTAGACCCCCTTTTAGAGCATCCGG-3′; and reverse, 5′-ATGCCTGCAGGTCGACGTCGATGGGAAAAGGCCTGA-3′. This PCR product was subcloned into the multicloning site between XbaI and SalI in the pmirGLO Dual-Luciferase miRNA Target Expression Vector (Promega, Madison, WI, USA), using the In-Fusion HD Cloning Kit (Takara Bio USA, Mountain View, CA, USA). The entire insert and vector sequence was confirmed by Sanger sequencing (Genome Information Research Center, The University of Osaka).

### 4.12. Luciferase Reporter Assay

HCT116 cells were seeded in 96-well plates, at 1 × 10^4^ cells per well, and transfected with 50 nM (final concentration) of either miR-NC (MISSION^®^ siRNA Universal Negative Control #1, SIC001-10NMOL; Sigma-Aldrich) or miR-136-5p (Ajinomoto Bio-Pharma), using Lipofectamine RNAiMAX. After 6 h, the cells were additionally transfected with 25 ng per well of the pmirGLO reporter vector, containing the DCLK1 3′ UTR fragment encompassing the predicted miR-136-5p binding site, using Lipofectamine 2000 (Thermo Fisher Scientific). At 12 h after plasmid transfection, luciferase activity was measured using the Dual-Luciferase Reporter Assay System (Promega), following the manufacturer’s instructions. Firefly luciferase activity was normalized to Renilla luciferase activity. Six independent transfection replicates were performed.

### 4.13. Tumor-Formation Ability Assay

ZsGreen^−^ and Enriched ZsGreen^+^ PANC-1 cells were resuspended in Matrigel Matrix (Corning, Corning, NY, USA), at a 1:1 ratio (*v*/*v*), and then subcutaneously injected into 8-week-old female SCID beige mice (CLEA Japan, Inc., Tokyo, Japan), with 150 cells per injection site (*n* = 3 mice per group). A low-cell-number transplantation design was used to assess tumor-initiating capacity under limiting conditions, rather than to maximize tumor take. This approach was based on our previous ZsGreen–ODC CSC model study, in which Enriched ZsGreen^+^ cells showed preferential tumor formation even at low inoculum sizes such as 10 or 100 cells [19]. In the present study, 150 cells were selected as a slightly less stringent but still limiting inoculum, under which tumor formation in ZsGreen^−^ cells could also have occurred. Nevertheless, tumors developed only in the Enriched ZsGreen^+^ group. Mice were monitored for tumor formation for up to 42 days. At the experimental end-point, or upon meeting humane end-point criteria, the mice were euthanized by cervical dislocation under deep anesthesia, performed by well-trained personnel.

### 4.14. In Vivo Xenograft Therapeutic Experiments

Enriched ZsGreen^+^ PANC-1 cells (5 × 10^5^ cells) were resuspended in Matrigel Matrix, at a 1:1 ratio (*v*/*v*), and then subcutaneously injected into both sides of the lower back regions of 8-week-old female nude mice (CLEA Japan, Inc.). No formal a priori sample size calculation was performed. Mice were randomly allocated to receive saline (*n* = 5), negative control miRNA (miR-NC; *n* = 5), or miR-136-5p (*n* = 5). No formal inclusion or exclusion criteria were established before the experiment, except for predefined humane endpoints. One mouse in the miR-136-5p group died before treatment initiation for reasons not considered treatment-related, yielding a final evaluable sample size of *n* = 4 for that group. miR-136-5p or miR-NC was formulated with sCA, as previously described [47,48]. Briefly, 4 µL of 1 M CaCl_2_ (FUJIFILM Wako Pure Chemical Corporation; 039-00475) was mixed with the miRNA in 1 mL of an inorganic solution containing 44 mM NaHCO_3_ (FUJIFILM Wako Pure Chemical Corporation; 191-01305) and 0.9 mM NaH_2_PO_4_·2H_2_O (FUJIFILM Wako Pure Chemical Corporation; 192-02815) at pH 7.5, followed by incubation at 37 °C for 30 min. The mixture was centrifuged at 12,000 rpm for 3 min, and the pellet was dissolved in saline (Otsuka Pharmaceutical Co., Ltd., Tokyo, Japan; 10095-6) containing 0.5% albumin prepared from Albuminar-25% Intravenous 12.5 g/50 mL (CSL Behring, Tokyo, Japan; 731003912). The formulation was then sonicated in a water bath for 10 min and intravenously administered within 10 min. On day 12 after implantation, intravenous administration via the tail vein was initiated, at a dose of 40 µg per injection, every 2–3 days, for a total of nine injections. Tumor volume was measured with calipers every 3–5 days, and calculated using the formula: (length × width^2^)/2. Investigators were not blinded to group allocation during treatment administration, tumor measurement, or data analysis. Tumors were harvested on day 24 after treatment initiation. All animal experiments were approved by the Institutional Animal Care and Use Committee of The University of Osaka (approval no. 13377-5, approved on 10 December 2019), and were conducted in accordance with institutional guidelines. Humane end-points were predefined, and applied when the maximum tumor diameter exceeded 15 mm, or when body weight loss exceeded 20%. At the experimental end-point, or upon meeting humane end-point criteria, the mice were euthanized by cervical dislocation under deep anesthesia, performed by well-trained personnel.

### 4.15. Statistical Analysis

Data are presented as mean ± SD unless otherwise indicated. Statistical analyses were performed using Microsoft Excel for Mac, version 16.107.3 (Microsoft Corporation, Redmond, WA, USA) and R version 4.5.2 (R Foundation for Statistical Computing, Vienna, Austria). Comparisons between two groups were performed using an unpaired two-tailed Welch’s *t*-test. For comparisons involving more than two groups at a single time-point (e.g., tumor weight at the end-point), one-way ANOVA was performed, followed by Dunnett’s multiple comparisons test versus the miR-NC group, using the multcomp package (version 1.4.30; single-step adjustment method). Longitudinal tumor volume data were analyzed using a linear mixed-effects model fitted with the lme4 package (version 2.0.1). Treatment group, day, and the Group × Day interaction were included as fixed effects. Random intercepts were specified for MouseID to account for repeated measurements within the same animal. Type III ANOVA was conducted using the lmerTest package (version 3.2.1). The primary effect of interest was the Group × Day interaction, which evaluates whether tumor growth trajectories differed among treatment groups over time. Post-hoc comparisons of tumor growth slopes were performed using estimated marginal means with the emmeans package in R (version 2.0.2) with Tukey adjustment. For NanoString nCounter analysis, the relative expression between ZsGreen^−^ and Enriched ZsGreen^+^ cells was assessed using an unpaired two-tailed Welch’s *t*-test. Given the limited number of biological replicates (*n* = 2 per group), this analysis was considered exploratory, and *p* values were not adjusted for multiple comparisons. A *p* value < 0.05 was considered statistically significant.

## Data Availability

The original contributions presented in this study are included in the article/Supplementary Materials. Further inquiries can be directed to the corresponding author.

## Supplementary Materials

The following supporting information can be downloaded at: https://www.mdpi.com/article/10.3390/ijms27083686/s1.
