# Supplementary figures and images for: miR-136-5p Preferentially Suppresses Cancer Stem-like Cells in Pancreatic Cancer
Source: Int J Mol Sci. 2026 Apr 21;27(8):3686. doi: 10.3390/ijms27083686 (PMC13116679; doi:10.3390/ijms27083686)

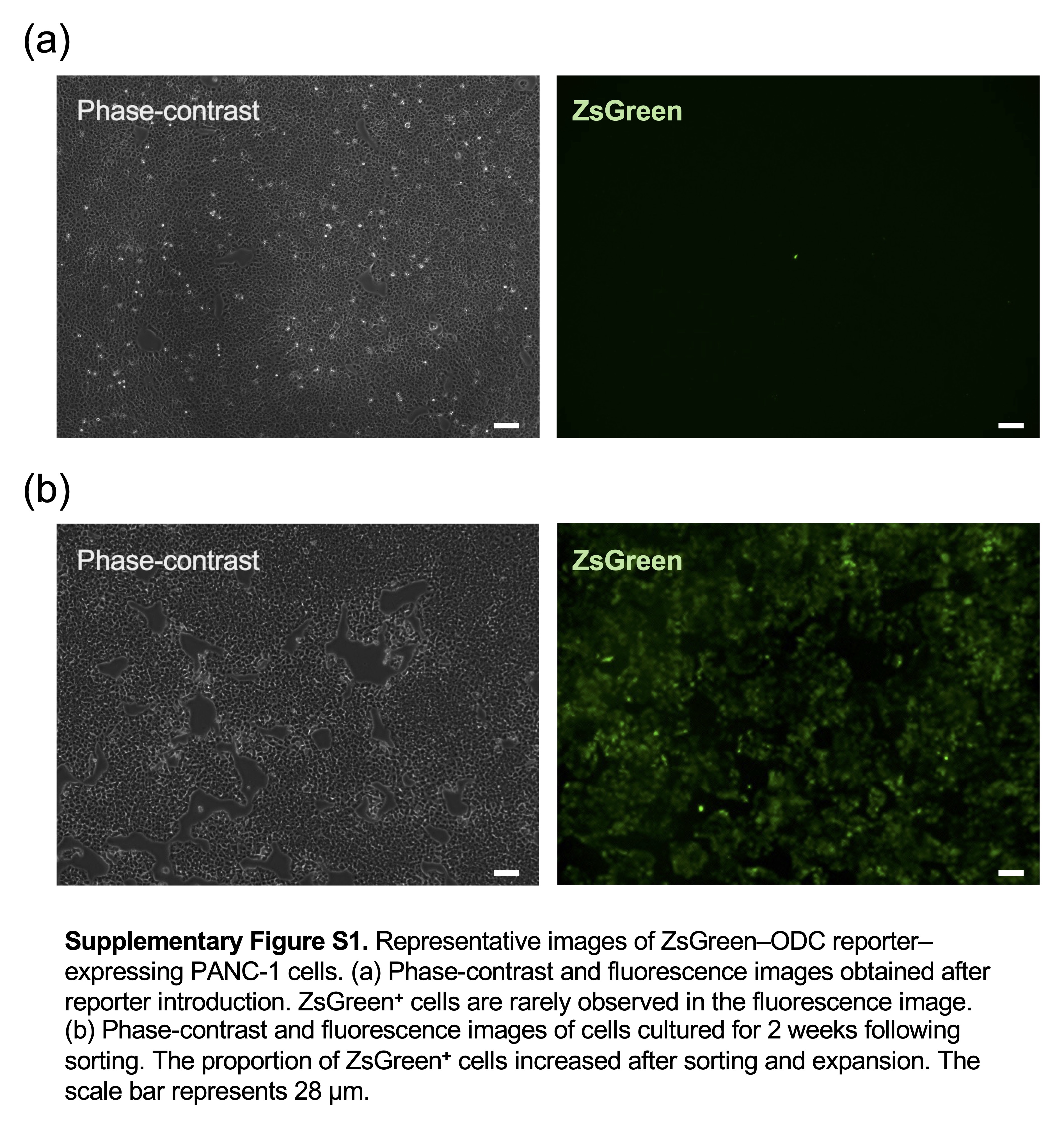

Supplement: Supplementary file 1 [file ijms-27-03686-s001.zip › Figure S1.jpg]

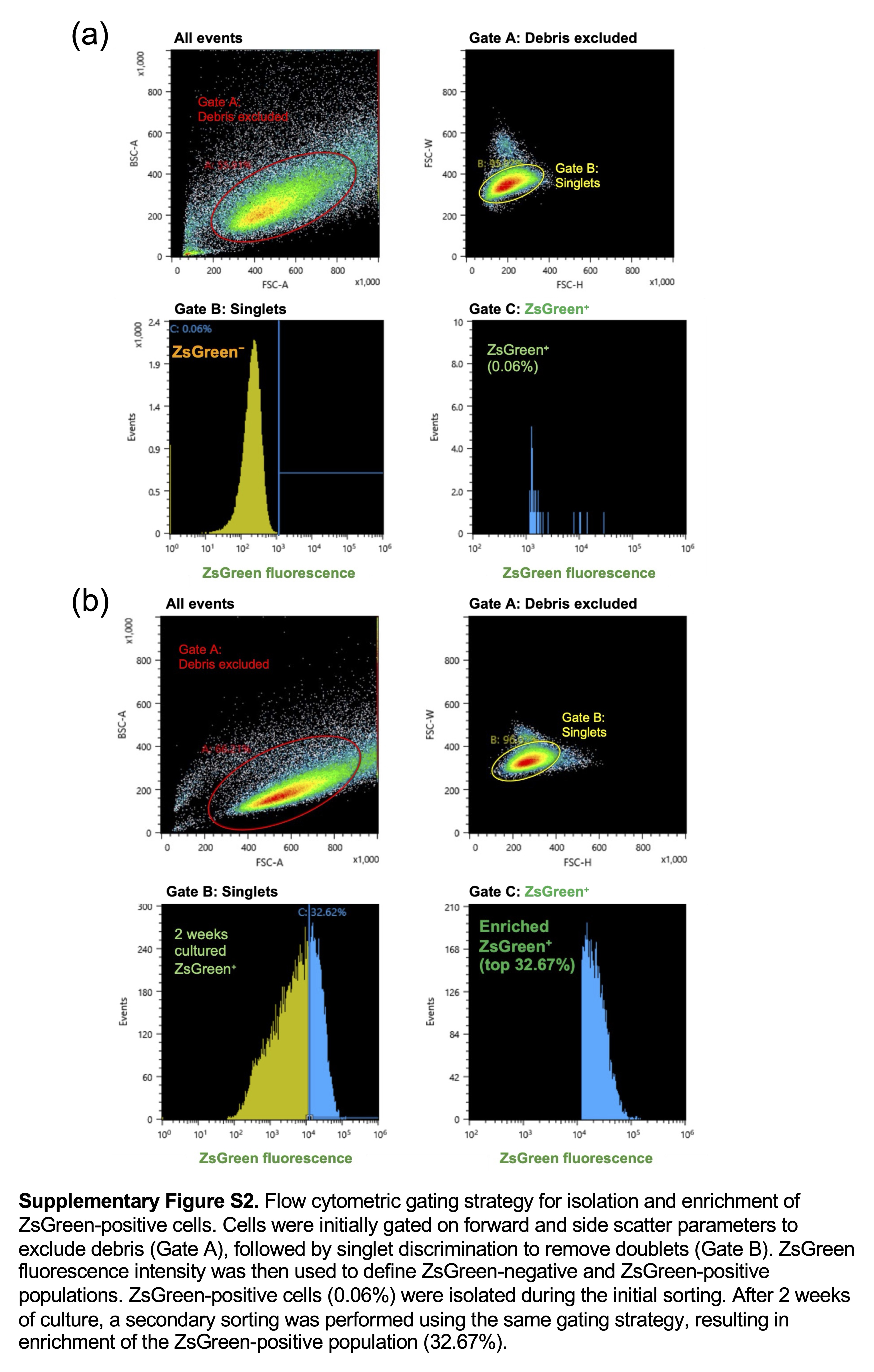

Supplement: Supplementary file 1 [file ijms-27-03686-s001.zip › Figure S2.jpg]

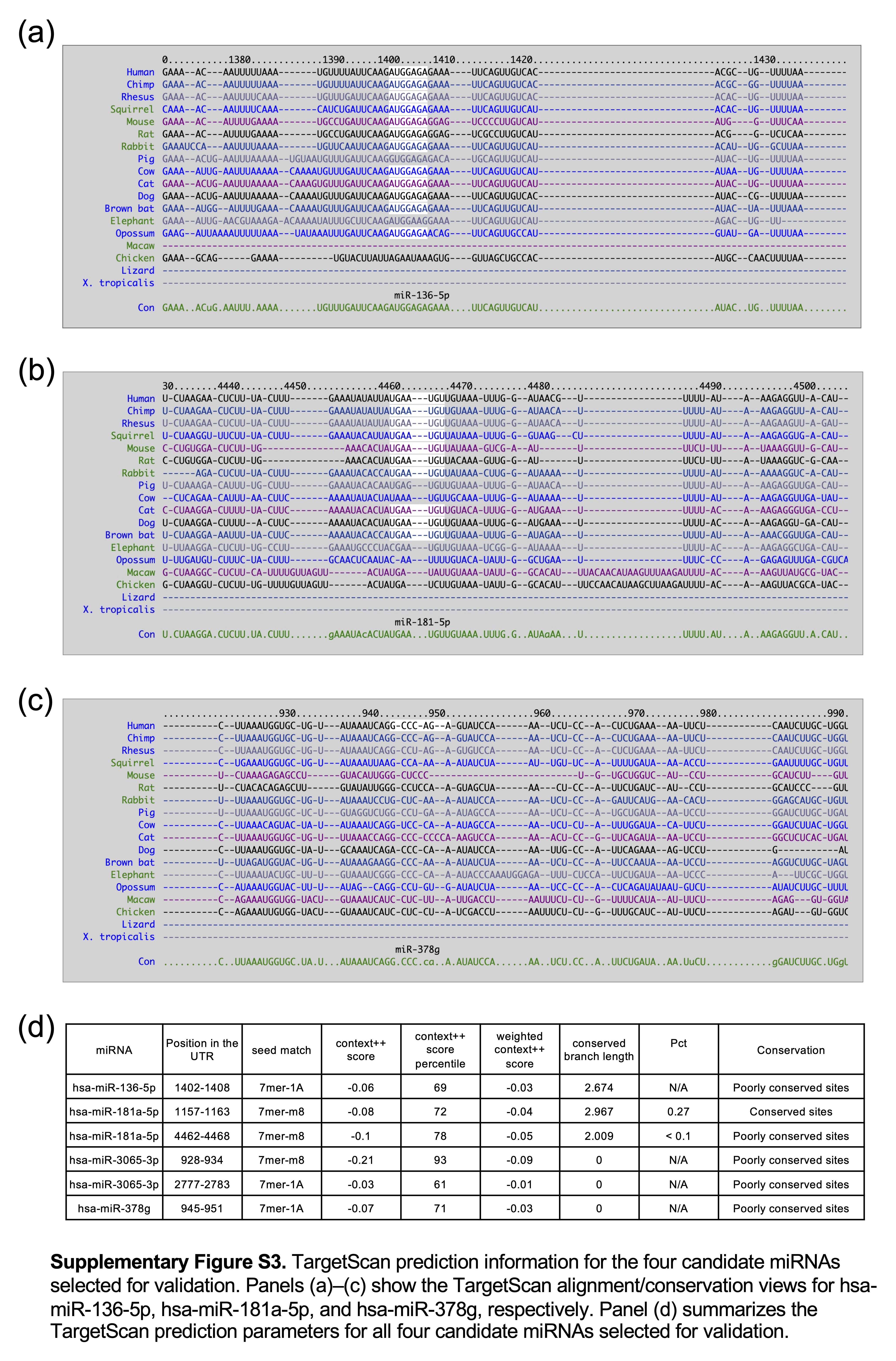

Supplement: Supplementary file 1 [file ijms-27-03686-s001.zip › Figure S3.jpg]

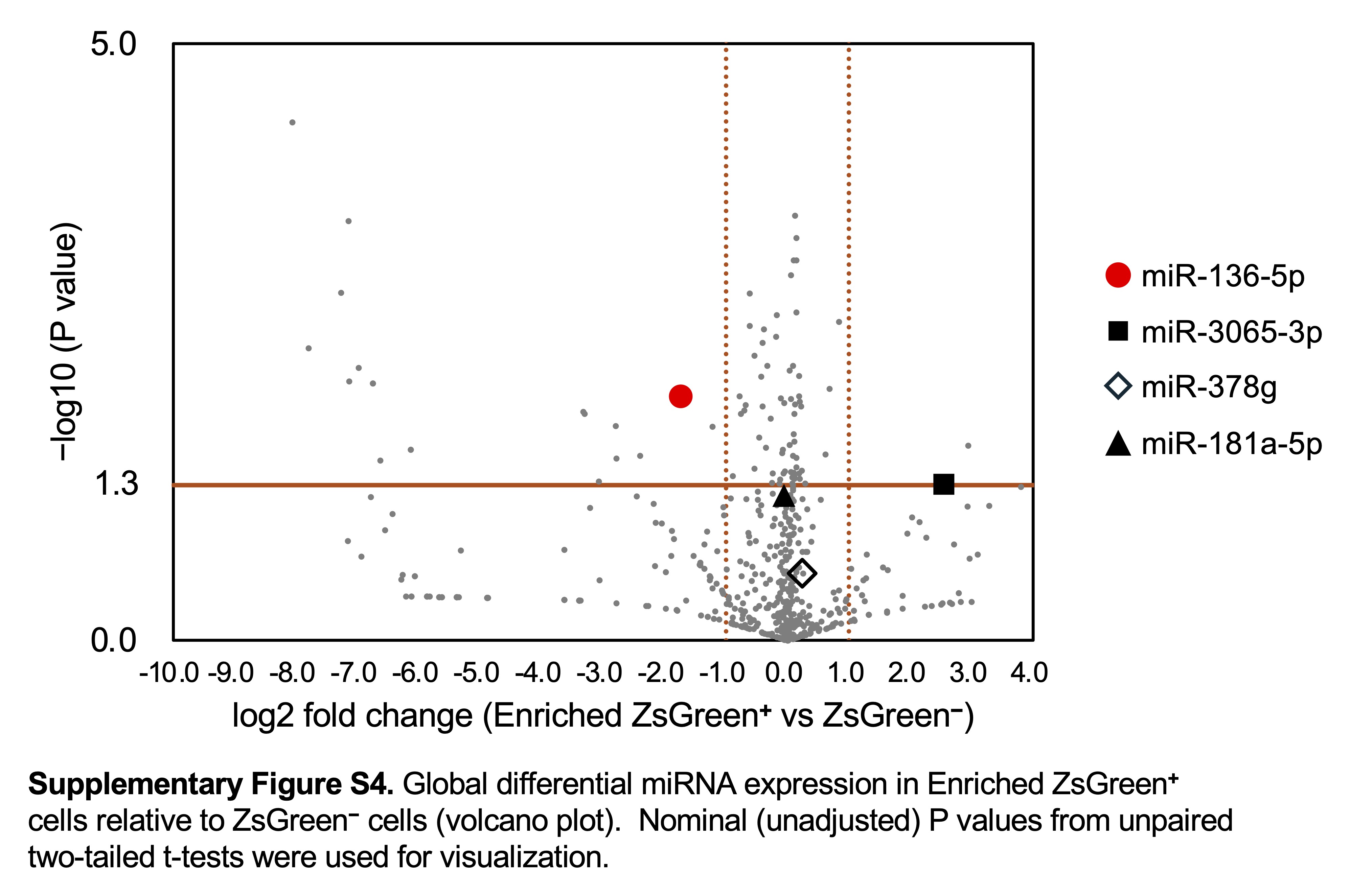

Supplement: Supplementary file 1 [file ijms-27-03686-s001.zip › Figure S4.jpg]

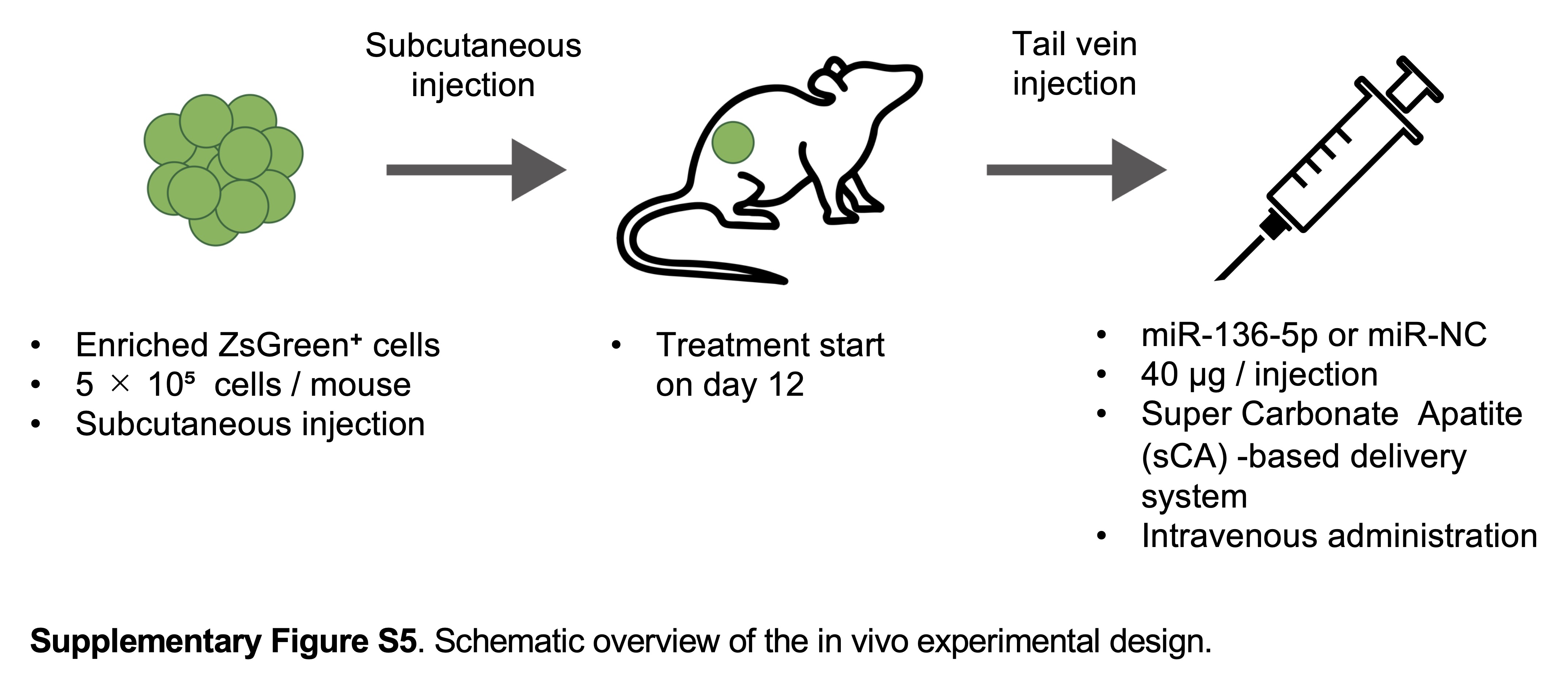

Supplement: Supplementary file 1 [file ijms-27-03686-s001.zip › Figure S5.jpg]

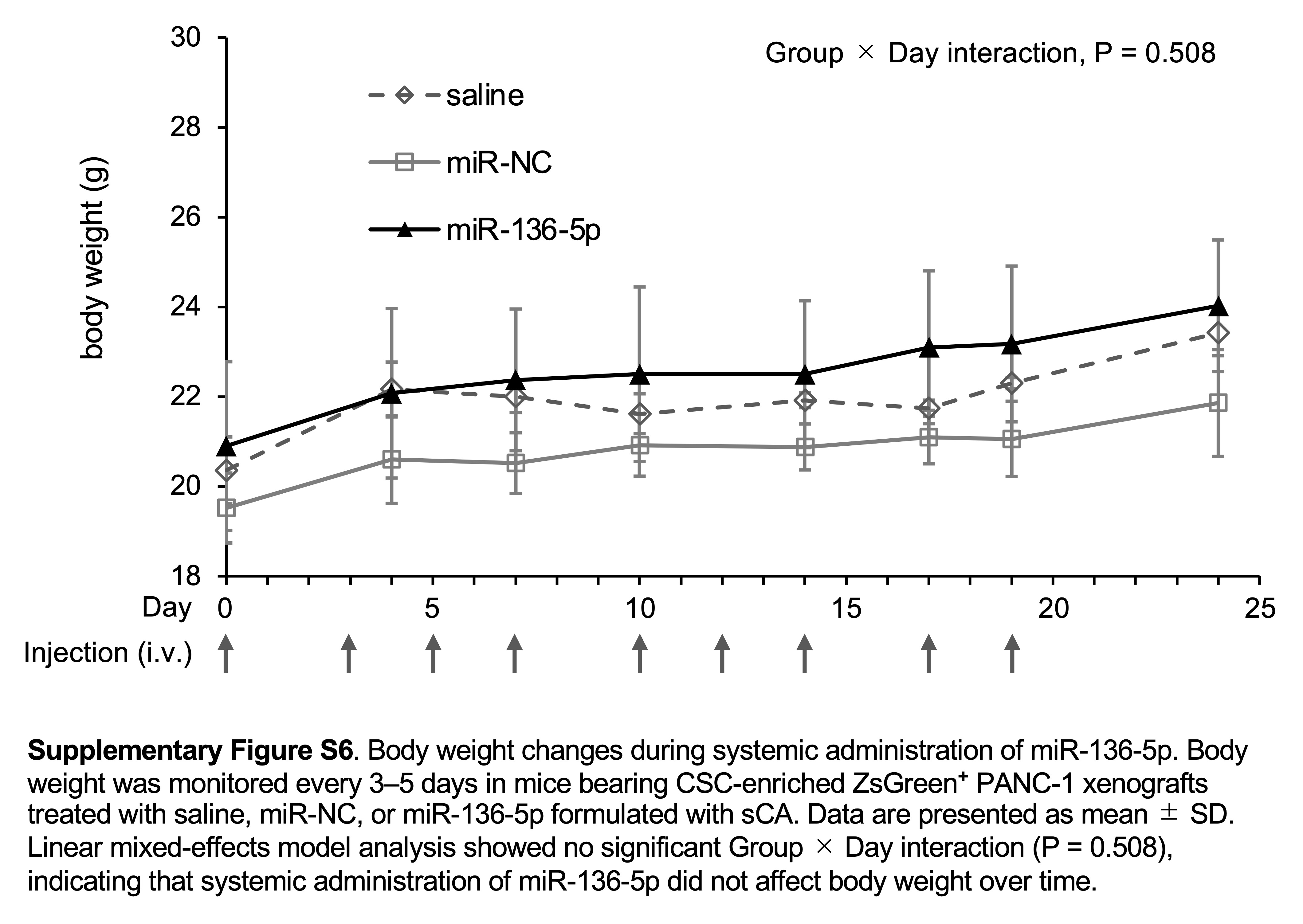

Supplement: Supplementary file 1 [file ijms-27-03686-s001.zip › Figure S6.jpg]

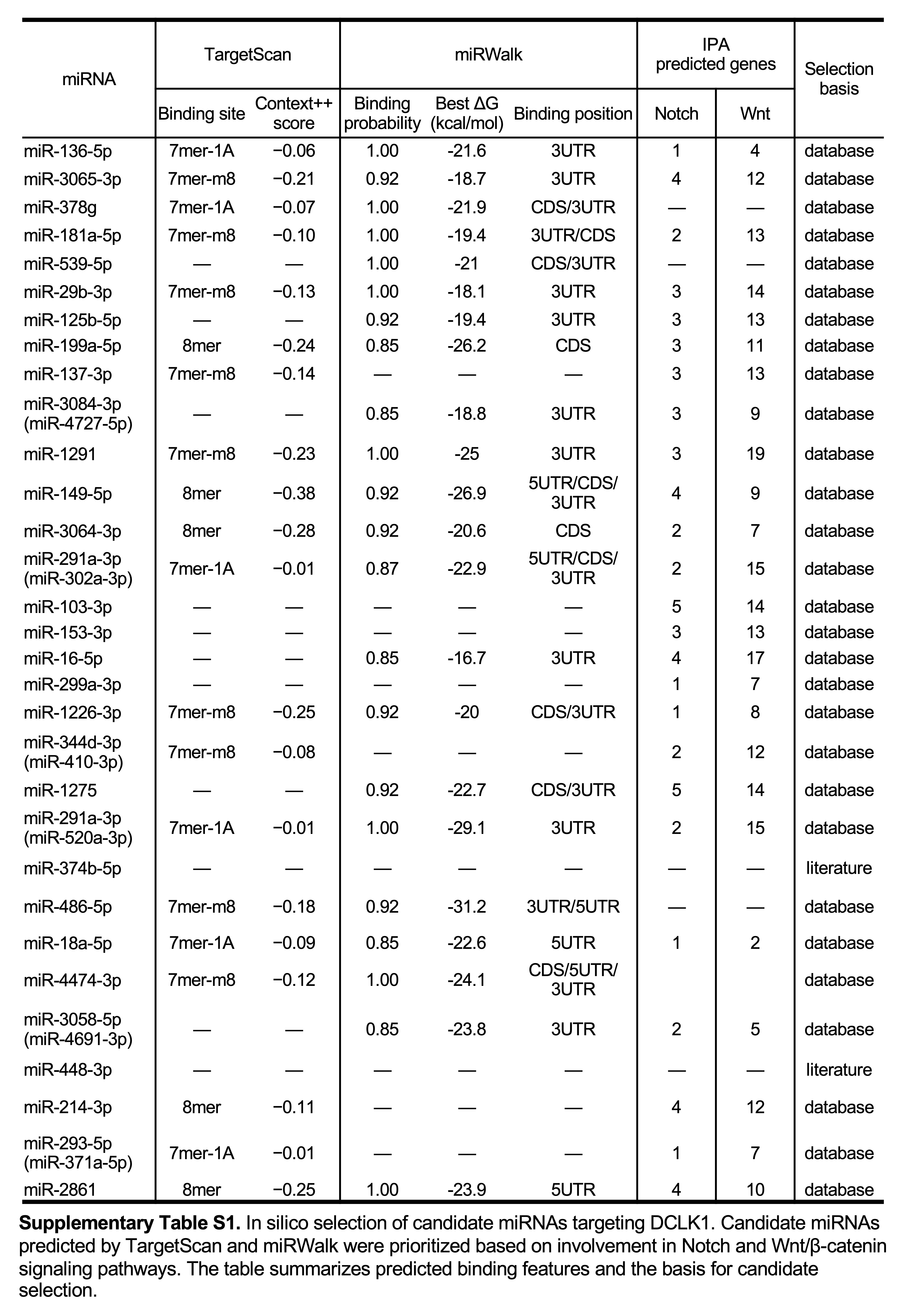

Supplement: Supplementary file 1 [file ijms-27-03686-s001.zip › Table S1.jpg]

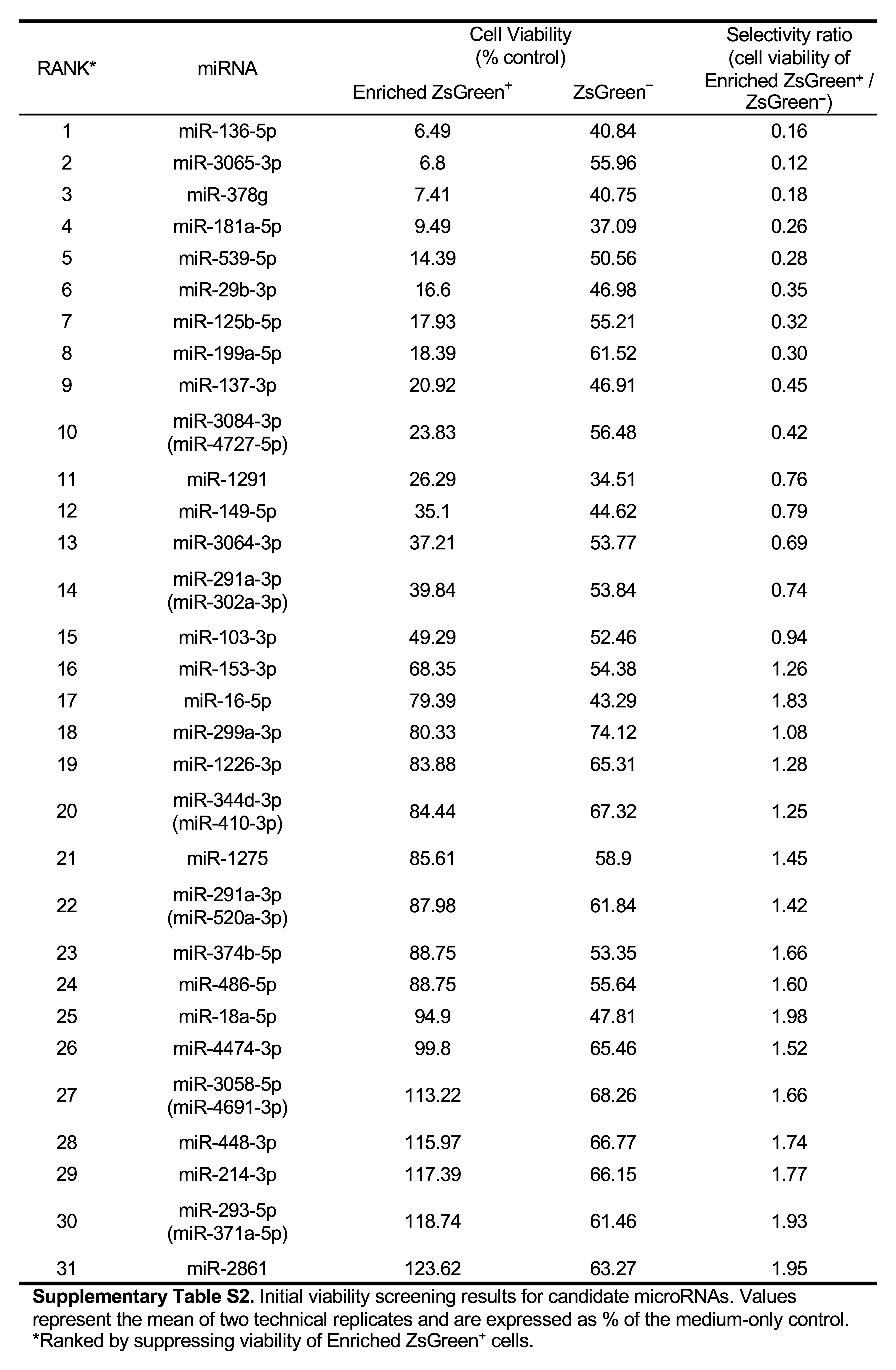

Supplement: Supplementary file 1 [file ijms-27-03686-s001.zip › Table S2.jpg]

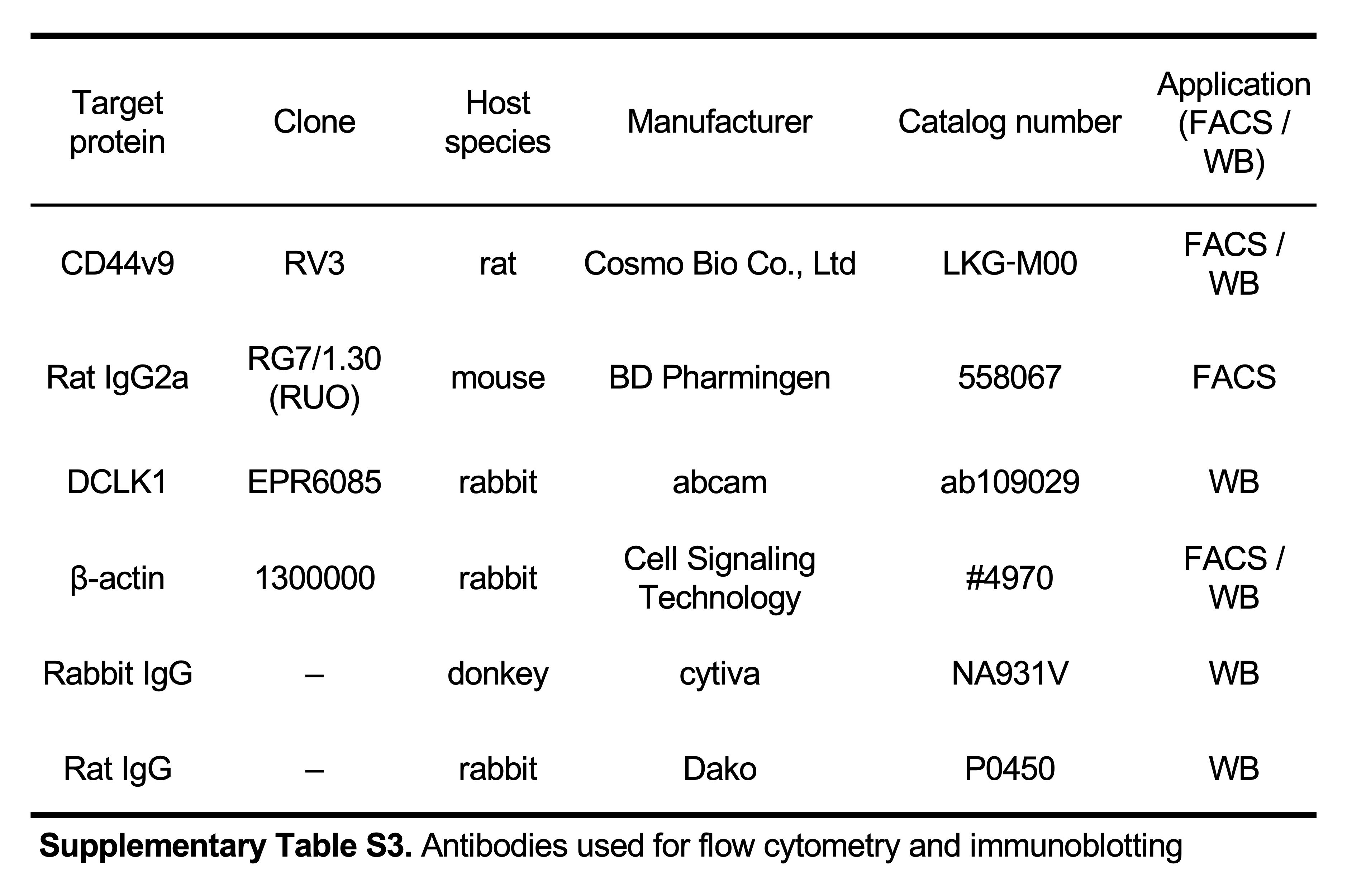

Supplement: Supplementary file 1 [file ijms-27-03686-s001.zip › Table S3.jpg]

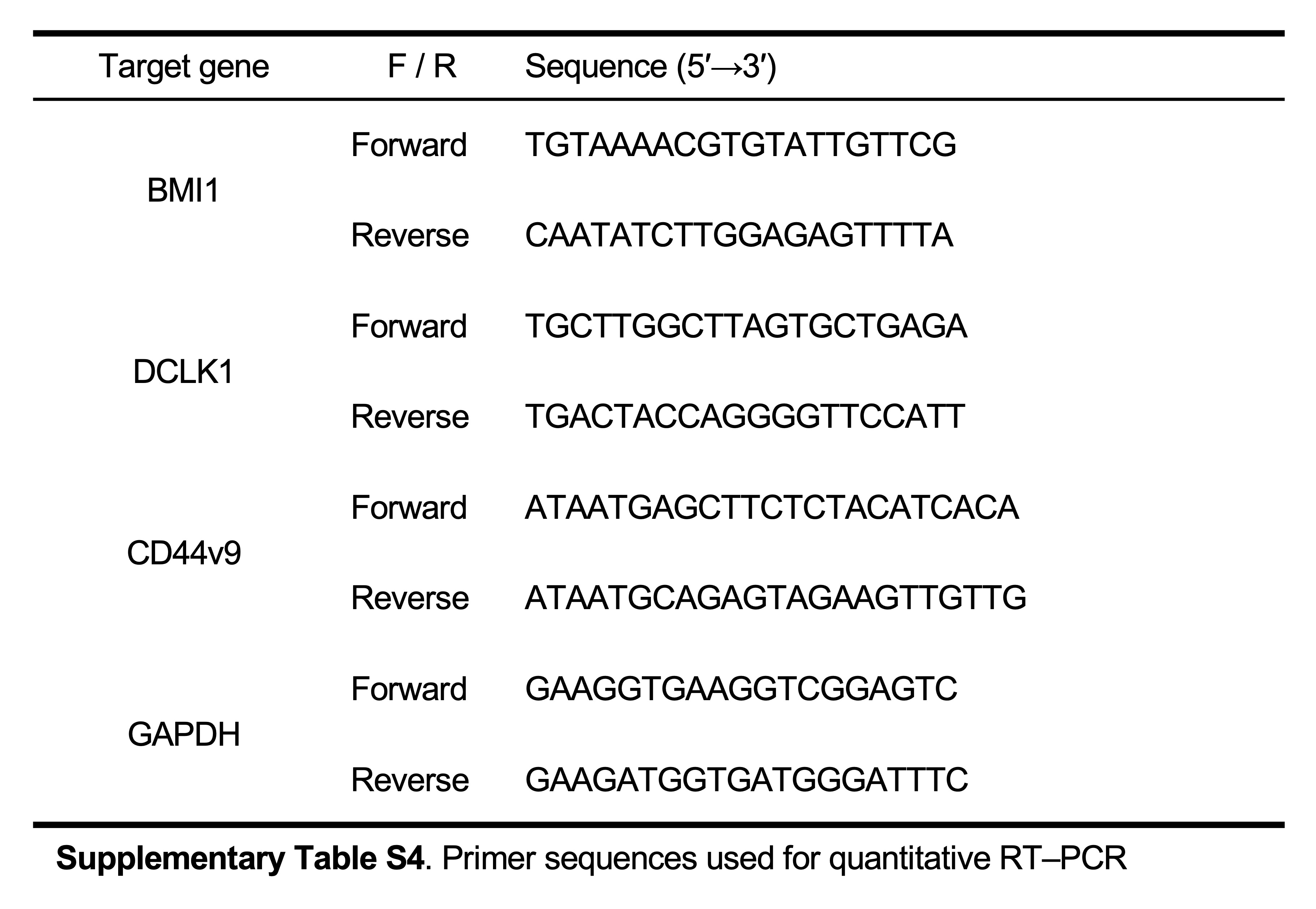

Supplement: Supplementary file 1 [file ijms-27-03686-s001.zip › Table S4.jpg]

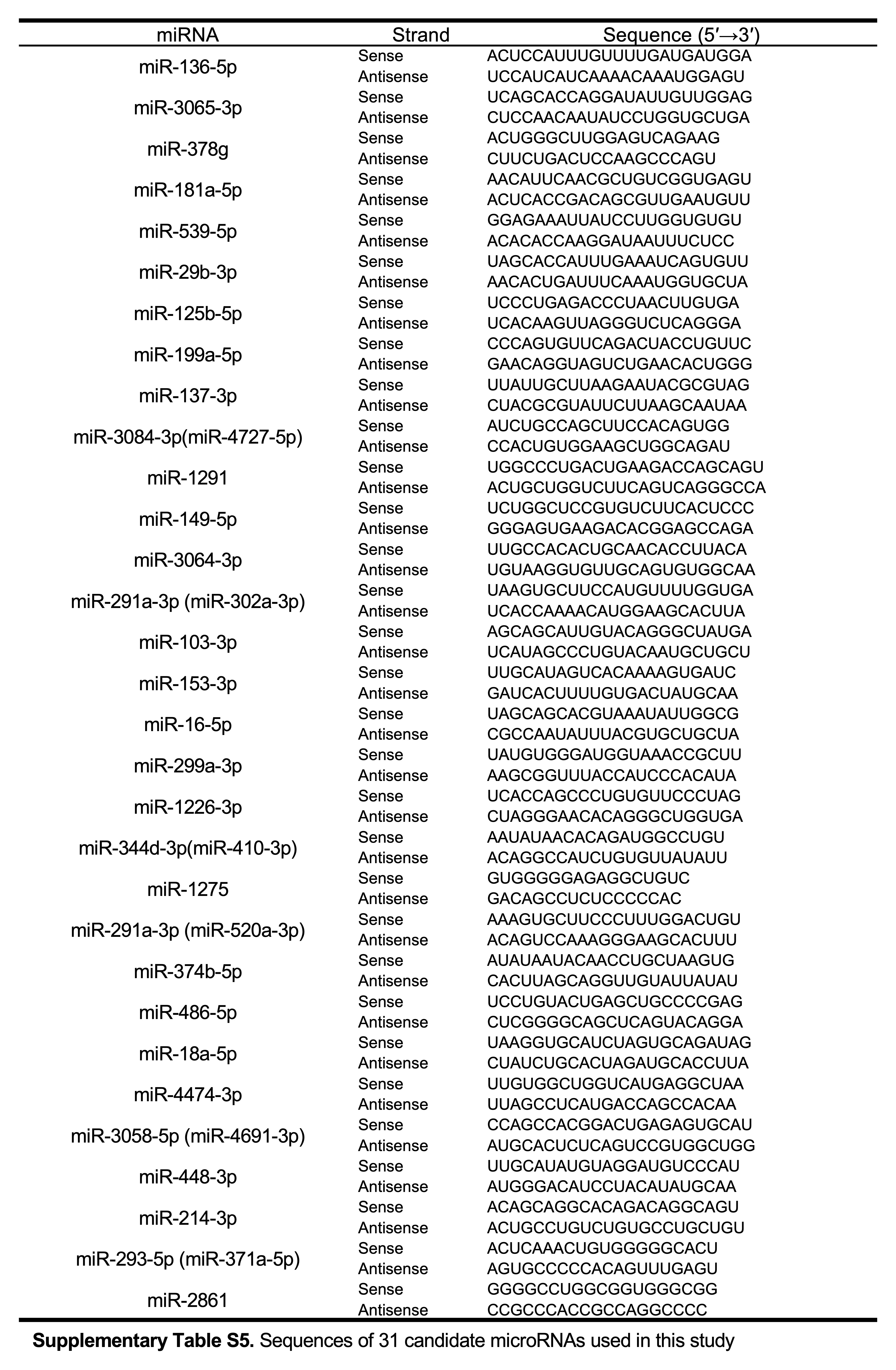

Supplement: Supplementary file 1 [file ijms-27-03686-s001.zip › Table S5.jpg]
